# Comparison of the Outcome of Conventional Osteosarcoma at Two Specialist International Orthopaedic Oncology Centres

**DOI:** 10.1080/13577140410001679202

**Published:** 2004-03

**Authors:** Samuel Ford, Adnan Saithna, Robert J. Grimer, Piero Picci

**Affiliations:** 1 Royal Orthopaedic Hospital Oncology Service Royal Orthopaedic Hospital Bristol Road South Birmingham B31 2AP UK; 2 The Istituto Ortopedico Rizzoli Bologna Italy

## Abstract

*Objective*: To determine the prognostic value of patient and treatment parameters in osteosarcoma, and whether these are equally important across international boundaries.

*Design*: Retrospective, cross-sectional study of 428 patients diagnosed with around-knee osteosarcoma, between 1990 and 1997 in Birmingham, UK, and Bologna, Italy. Disease-free survival (DFS) and overall survival (OS) assessed by Kaplan–Meier, Fisher's PLSD and Cox proportional hazard regression.

*Results*: Five-year DFS and OS were 56 and 73% at Centre 1, compared to 43 and 60% at Centre 2 (*P*=0.0022 and *P* = 0.025, respectively). The most important bad prognostic factors for DFS and OS respectively were raised alkaline phosphatase at diagnosis (*P*=0.002 and *P*=0.003), tumour necrosis < 90% following chemotherapy (*P*=0.001 and *P* = 0.004) and volume > 150 cm^3^ at diagnosis (*P*=0.04 and *P*=0.006). The most significant combination of bad prognostic factors was alkaline phosphatase and tumour necrosis. A total of 73% of patients at Centre 1 had greater than 90% necrosis of the tumour following neoadjuvant chemotherapy compared with 29% at Centre 2.

*Conclusions*: Tumour-based prognostic factors have similar significance across international boundaries. Chemotherapy effectiveness appears to be a major factor in explaining the survival difference between the two centres.


*Sarcoma*, March 2004, *VOL. 8, NO. 1, 13–18*

Taylor & Francis healthsciences

ORIGINAL ARTICLE

### Comparison of the outcome of conventional osteosarcoma at two specialist international orthopaedic oncology centres

SAMUEL FORD[1], ADNAN SAITHNA[1], ROBERT J. GRIMER[1] & PIERO PICCI[2]

[1]*The Royal Orthopaedic Hospital, Birmingham, UK &* [2]*The Istituto Ortopedico Rizzoli, Bologna, Italy*

**Abstract**

*Objective*: To determine the prognostic value of patient and treatment parameters in osteosarcoma, and whether these are equally important across international boundaries.

*Design*: Retrospective, cross-sectional study of 428 patients diagnosed with around-knee osteosarcoma, between 1990 and 1997 in Birmingham, UK, and Bologna, Italy. Disease-free survival (DFS) and overall survival (OS) assessed by Kaplan–Meier, Fisher's PLSD and Cox proportional hazard regression.

*Results*: Five-year DFS and OS were 56 and 73% at Centre 1, compared to 43 and 60% at Centre 2 ($P = 0.0022$ and $P = 0.025$, respectively). The most important bad prognostic factors for DFS and OS respectively were raised alkaline phosphatase at diagnosis ($P = 0.002$ and $P = 0.003$), tumour necrosis < 90% following chemotherapy ($P = 0.001$ and $P = 0.004$) and volume >150 $cm^3$ at diagnosis ($P = 0.04$ and $P = 0.006$). The most significant combination of bad prognostic factors was alkaline phosphatase and tumour necrosis. A total of 73% of patients at Centre 1 had greater than 90% necrosis of the tumour following neoadjuvant chemotherapy compared with 29% at Centre 2.

*Conclusions*: Tumour-based prognostic factors have similar significance across international boundaries. Chemotherapy effectiveness appears to be a major factor in explaining the survival difference between the two centres.

**Key words:** *osteosarcoma, prognostic factors, survival*

### Introduction

Chemotherapy has dramatically changed the prognosis for patients with osteosarcoma. The optimum regime has yet to be determined and few randomised clinical trials have helped clarify the problem, often showing little difference in outcome between regimes.[1–6]

A large number of clinical studies have attempted to identify the prognostic factors that influence survival in osteosarcoma. However, variation in methodology has led to inconsistent results and difficulty in interpreting the true prognostic effect of many of the variables evaluated.[7–13] In any case, most of the prognosticators reported lack specificity as pre-treatment predictive factors, thus preventing their use as a basis for a more differentiated algorithm of risk-adapted chemotherapeutic and surgical intervention. Furthermore, few studies have looked at whether these prognostic factors are equally pertinent in different therapeutic and geographical populations. This paper therefore attempts to identify the importance of some of the prognosticators already reported in the literature and whether they hold equal value at two specialist orthopaedic oncology centres from different countries.

A second factor was trying to identify whether there was any evidence for a perceived difference in survival between patients treated at the two centres and to find potential reasons for this.

### Methods

The two centres used for this study are both large centres of musculoskeletal oncology and both maintain prospective databases of patients treated, their treatment and outcome. Both centres agreed that their blinded data could be collected and used for this study without wishing to identify specifically which centre was which. Hence the centres will hereafter be identified as Centre 1 and Centre 2.

A computer database search was used to identify all patients under the age of 40 who were diagnosed with conventional high grade osteosarcoma localised to the distal femur or proximal tibia between 1990

Correspondence to: R.J. Grimer, Royal Orthopaedic Hospital Oncology Service, Royal Orthopaedic Hospital, Bristol Road South, Birmingham B31 2AP, UK. Tel.: +44-121-685-4150; Fax: +44-121-685-4146; E-mail: rob.grimer@roh.nhs.uk

1357-714X print/1369–1643 © 2004 Taylor & Francis Ltd
DOI: 10.1080/13577140410001679202

and 1997 at the two study centres. Patients with skip lesions or metastatic disease at diagnosis were excluded.

Individual patient records were traced where possible. The patient and treatment parameters recorded are listed in Table 1. A total of nine different chemotherapy regimes were used between the two centres. Comparisons between these regimes were outside the remit of this study and have already been published elsewhere.[1,14–16] Hence, we merely compared chemotherapy at centre 1 with that used at centre 2.

The data were analysed using the 'Statview' statistical analysis program with respect to disease-free survival (DFS) (time in weeks to developing metastatic disease and/or local recurrence) and overall survival (OS) (time in weeks to death from date of diagnosis).[2] All data were analysed using Kaplan–Meier survival analyses with the exception of excision margins and histological subtype which were analysed using Fisher's PLSD ANOVA post-hoc test. Survival curves, generated by Kaplan–Meier analysis, were compared with the log rank test and significance was set at $P < 0.05$. Significant parameters were further analysed using the Cox proportional hazards model for multivariate analysis. Hazard ratios have been calculated using a proportional hazards method with only the noted covariate in the model. Chi-squared and $P$ values generated were used to denote co-variation.

**Table 1.** *Patient and treatment parameters recorded*

| |
|---|
| Specialist centre at which treatment received |
| Patient gender |
| Age at diagnosis |
| Date of diagnosis |
| Symptom duration |
| History of trauma |
| Presence of pathological fracture at diagnosis |
| Serum alkaline phosphatase level at diagnosis |
| Tumour volume ($cm^3$)* |
| Chemotherapy regime employed |
| Surgical technique |
| Histological subtype |
| Percentage chemotherapy induced necrosis |
| Enneking Stage at diagnosis |
| Tumour margins |
| Date of last follow-up |
| Status at last follow-up (dead, dead from a non-correlated cause, alive and disease free, alive with metastases, alive with local recurrence, alive with metastatic disease and local recurrence) |
| Time from diagnosis to death/metastatic disease/local recurrence |

*Volume was calculated by using the formulas: $A \times B \times C \times 0.52$ for spherical tumours and $A \times B \times C \times 0.735$ for elliptical tumours, where $A$, $B$ and $C$ are the three dimensions of the tumour.

### Results

*Population demographics*

The study population comprised 428 patients, 265 patients from centre 1 and 163 from centre 2. There was a male preponderance with a gender ratio of 1.4:1 and the average age at diagnosis was 15.8 years. There were 271 tumours of the distal femur and 157 of the proximal tibia. The mean duration of symptoms prior to diagnosis was 14 weeks (median 8 weeks). Twenty-one patients had a pathological fracture. The mean diameter of the tumours was 10 cm and the volume was 239 ml. Table 1 lists the factors studied and Table 2 shows the proportion of patients with different factors at each centre.

*Treatment variables*

All patients underwent attempts at curative treatment with chemotherapy and surgery. The margins of excision were documented in 411 patients and were intralesional in 15, marginal in 54, wide in 324 and radical in 18 (this only arose following amputation). Adequate margins were those found to be wide or radical. The percentage necrosis following neoadjuvant chemotherapy was available for 377 patients. Of these, 211 had a good response with > 90% necrosis, whilst 166 had a poor response with < 90%.

**Table 2.** *Variation in patient and treatment factors between the two centres*

| | Centre 1 | Centre 2 | Significance |
|---|---|---|---|
| *Patient factors* | | | |
| Mean age | 15.2 | 16.1 | NS |
| Mean duration of symptoms (weeks) | 11 | 19 | 0.01 |
| Sex ratio F/M | 105/160 | 75/88 | NS |
| Pathological fracture | 10/265 (3.7%) | 11/163 (6.7%) | NS |
| Raised alkaline phosphatase at diagnosis | 99/230 (43%) | 66/120 (55%) | 0.03 |
| Tibia/femur ratio | 94/171 | 63/100 | NS |
| Mean volume (ml) | 224 | 275 | NS |
| *Treatment factors* | | | |
| Mean percent necrosis | 89% | 62% | < 0.0001 |
| Proportion with > 90% necrosis | 169/232 (73%) | 42/145 (29%) | < 0.0001 |
| Proportion with inadequate margins | 19/259 (7%) | 50/152 (33%) | < 0.0001 |

Differences between the two centres are shown in Table 2.

*Local control*

Thirty-two patients developed a local recurrence. It arose in 24 of the 271 patients with distal femoral lesions (8.8%) and eight of the 156 with proximal tibial lesions (5.1%). Local recurrence was strongly related to margins of excision and effectiveness of chemotherapy. (Table 3)

*Patient survival*

Patients treated at Centre 2 were less successful in terms of DFS and OS. Five-year DFS was 56% in centre 1 and 43% in centre 2 ($P = 0.0022$) (Fig. 1). Five-year OS was 73% (CI ± 3%) in centre 1 and 60% (CI ± 4%) in centre 2 ($P = 0.025$) (Fig. 2). The effect of chemotherapy necrosis on survival at the different centres is similar but patients with poor necrosis at Centre 2 (5-year OS 57%) did a lot worse than those at Centre 1 (5-year OS 62%) (Fig. 3).

**Table 3.** *Showing the incidence of local recurrence in relation to both margins of excision and effectiveness of chemotherapy*

| | < 90% necrosis | > 90% necrosis |
|---|---|---|
| Intralesional or marginal margin | 7/37 (19%) | 3/19 (17%) |
| Wide or radical margin | 13/121 (11%) | 4/187 (2%) |

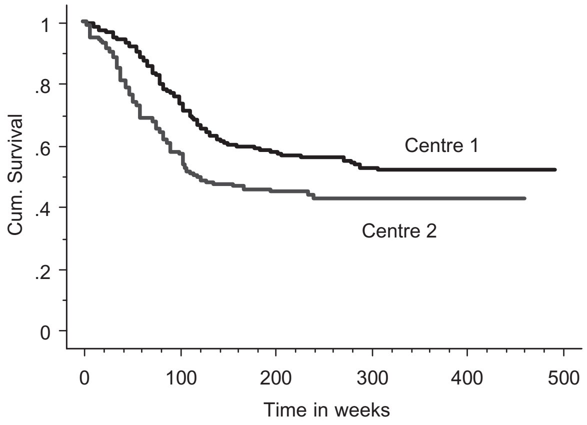

**Fig. 1.** *Kaplan–Meier analysis comparing DFS time for Centre 1 and Centre 2. Mantel–Cox P = 0.0022.*

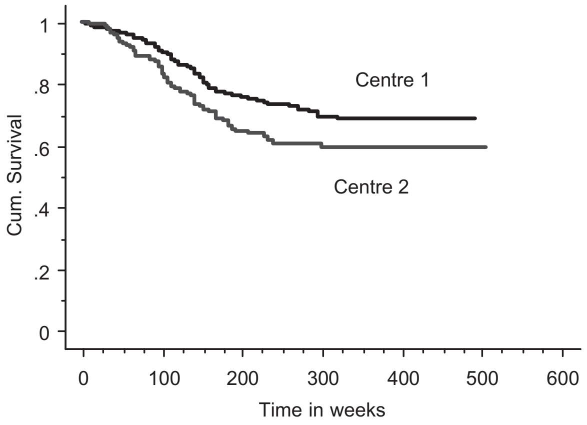

**Fig. 2.** *Kaplan–Meier analysis comparing OS for Centre 1 and Centre 2. Mantel–Cox P = 0.025.*

*Prognostic factors*

Tables 4 and 5 list patient and treatment parameters investigated by survival analysis with hazard ratios and confidence intervals generated using the Mantel Cox test.

Tumour volume greater than 150 $cm^3$, percentage chemotherapy induced necrosis less than 90%, and raised alkaline phosphatase at diagnosis are strongly significant for an association with less favourable DFS and OS. Gender, site, duration of symptoms, history of trauma, surgical stage, adequacy of surgical margins and presence of a pathological fracture at diagnosis were all shown to have no correlation with DFS or OS. There was no particular histological subtype shown to have any influence on OS.

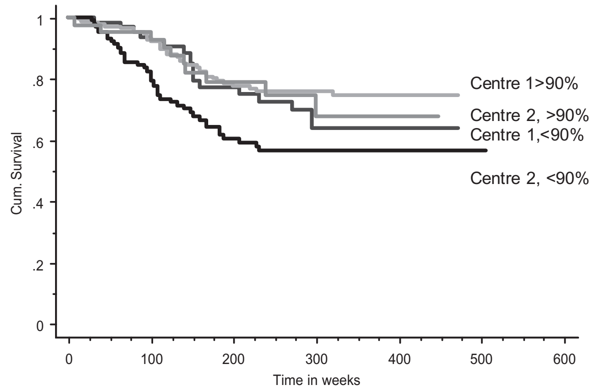

**Fig. 3.** *Kaplan–Meier analysis comparing overall survival for patients at Centres 1 and 2 split by percent necrosis.*

**Table 4.** *Details of factors affecting disease-free survival on univariate and multivariate analysis. A high hazard ratio implies an increased risk to overall survival in the presence of that factor, a lower ratio (< 1) implies a risk less than unity*

| | Hazard ratio | 95% CI | *P* value |
|---|---|---|---|
| *Factor* | | | |
| Symptoms < 8 weeks | 0.94 | 0.70–1.26 | 0.68 |
| Age < 16 | 1.26 | 0.95–1.66 | 0.10 |
| Size < 10 cm | 0.56 | 0.35–0.89 | 0.014 |
| Raised alkaline phosphatase | 1.61 | 1.19–2.19 | 0.002 |
| Volume >150 ml | 1.51 | 1.02–2.2 | 0.042 |
| Site: femur | 1.05 | 0.79–1.39 | 0.73 |
| Pathological fracture: | 0.78 | 0.39–1.59 | 0.49 |
| Sex: female | 0.96 | 0.73–1.27 | 0.78 |
| Necrosis < 90% | 1.62 | 1.21–2.17 | 0.0013 |
| Centre 2 | 1.54 | 1.17–2.02 | 0.0022 |
| Adequate surgical margins | 0.73 | 0.51–1.05 | 0.088 |
| *On multivariate analysis the following remained significant:* | | | |
| Necrosis <90% | 1.624 | 1.185–2.226 | 0.0026 |
| Raised alkaline phosphatase | 1.502 | 1.094–2.060 | 0.0117 |

**Table 5.** *Details of factors affecting overall survival on univariate and multivatiate analysis. A high hazard ratio implies an increased risk to overall survival in the presence of that factor, a lower ratio (< 1) implies a risk less than unity*

| | Hazard ratio | 95% CI | *P* value |
|---|---|---|---|
| *Factor* | | | |
| Symptoms <8 weeks | 1.06 | 0.74–1.53 | 0.74 |
| Age < 16 | 1.11 | 0.784–1.57 | 0.56 |
| Size < 10 cm | 0.65 | 0.36–1.15 | 0.14 |
| Raised alkaline phosphatase | 1.62 | 1.23–2.69 | 0.0027 |
| Volume >150 ml | 2.08 | 1.23–3.5 | 0.0057 |
| Site: femur | 1.07 | 0.75–1.53 | 0.71 |
| Pathological fracture: | 0.933 | 0.41–2.12 | 0.87 |
| Sex: female | 0.904 | 0.64–1.28 | 0.57 |
| Necrosis < 90% | 1.746 | 1.19–2.56 | 0.0044 |
| Centre 1 | 0.67 | 0.474–0.952 | 0.025 |
| Adequate surgical margins | 0.78 | 0.49–1.23 | 0.28 |
| No local recurrence | 0.54 | 0.32–0.94 | 0.029 |
| *On multivariate analysis the following remained significant:* | | | |
| Necrosis < 90% | 1.815 | 1.20–2.74 | 0.0044 |
| Raised alkaline phosphatase | 1.66 | 1.09–2.51 | 0.0165 |

Centre of treatment has a weak association with improved survival.

### *Multivariate analysis using the Cox model*

Factors found to be prognostically significant on univariate testing were systematically combined in an attempt to generate the highest total chi-squared value with all prognostic factors still reaching significance, this was deemed the combination of prognostic factors which best explains DFS and OS.

The combination found to best explains DFS and OS is alkaline phosphatase level at diagnosis and degree of chemotherapy induced necrosis (Tables 4 and 5).

### Discussion

The aim of this paper has been to determine the prognostic value of patient and treatment parameters in osteosarcoma and whether these are equally important across international boundaries. Secondly, to try and identify if there is a difference in outcome for patients with osteosarcoma between two centres and, if so, why? We have used prognostic factors previously found to be important and which could be readily collected from each centre.[9] We have specifically restricted this survey to tumours of the distal femur or proximal tibia as all authors agree that these two sites have similar outcomes.

Local recurrence is generally accepted to be a poor prognostic indicator.[18,19] We have confirmed it's relationship to both margins of excision and to effectiveness of chemotherapy. If either of these are unsatisfactory then the risks of local recurrence increase.[20–22] Like others, we have been unable to show a demonstrable and independent effect of LR on overall survival when analysed using multifactorial methods.

### *Age and gender*

Gender is not significantly correlated with adverse outcome. A male preponderance and average age at diagnosis of 15.8 years are consistent with data from other centres.[7]

### *Enneking stage at diagnosis*

Tumour stage holds no significance for DFS or OS. Lack of survival significance between stages could indicate that confinement within or invasion beyond the cortex has no prognostic significance; however, a lack of discriminating power of this classification may play a role in masking any trend.[23]

### *Histological subtype*

We were unable to confirm a recent report that histological subtype has any prognostic value.[24] Confounding factors in the analysis included inherent observer bias and small numbers of certain subtypes limiting statistical power.

### *Anatomical site*

Location of the tumour, be it distal femur or proximal tibia, has no bearing on DFS or OS. Similarly, a number of other authors have not found anatomical site to be a significant predictor of disease outcome.[12,14]

### *Tumour volume and size*

Tumours > 150 $cm^3$ are highly significant for a reduced DFS and OS lending status as a major prognostic factor in the pre-treatment assessment of new patients. Furthermore, tumour volume when considered as a continuous variable using the Cox model, remains significant for OS per unit increase in volume. Tumour size (maximum length) was not found to be useful in predicting OS or DFS.

### *Symptom duration*

We found greater symptom duration to be inversely correlated with DFS, with little influence on OS. Others have found the reverse or no association with a favourable outcome.[25] Sources of bias in this observation include symptom duration not being recorded for all patients and an element of recall bias on behalf of the patient.

*Alkaline phosphatase*

A raised serum alkaline phosphatase at diagnosis is highly correlated with shorter DFS and OS.

Interestingly serum alkaline phosphatase given as normal or raised does not carry the same degree of significance for a shorter DFS or OS in centre 2 as that demonstrated in centre 1. This may be because the mean percentage necrosis for centre 2 is significantly lower than that for centre 1 with the strong correlation between percentage necrosis and DFS and OS partially masking the contribution of alkaline phosphatase as a determinator of disease recurrence and poor survival. Furthermore when tumour volume and alkaline phosphatase are analysed simultaneously by multivariate analysis, $P$ values rise and chi-squared values drop indicating a degree of co-variation between tumour volume and raised alkaline phosphatase. A possible explanation would be that tumours capable of producing alkaline phosphatase manufacture increasingly larger quantities as the tumour burden increases. This would be consistent with reports that alkaline phosphatase is more likely to be elevated in those presenting with metastatic disease.[11] However, tumours that do not produce alkaline phosphatase may be just as advanced as those that do. An alkaline phosphatase-producing tumour could be more aggressive, with a relative resistance to chemotherapy induced necrosis. However, this is unlikely as we could find no correlation between alkaline phosphatase and percentage necrosis in the Cox model.

*Degree of necrosis*

Degree of chemotherapy induced necrosis has long been recognised as the most important predictor of subsequent outcome for localised extremity disease.[26] A greater degree of necrosis incurs a more favourable outcome. Micrometastases from therapeutically sensitive primaries are logically less likely to remain viable than those of resistant lesions.

Percentage necrosis is significantly greater in centre 1 than centre 2 and has greater significance in centre 2 for predicting adverse outcome. Could percentage necrosis account for the survival discrepancy between the centres? The observation that 73% of patients in Centre 1 appear to have a good response to chemotherapy compared with just 29% in Centre 2 probably goes a long way to explaining the survival difference between the two centres. However, even patients with a good response at Centre 2 do not do as well as those at Centre 1 (Fig. 3) and the reason for this is not clear from this study. One possible reason for the difference in degree of chemotherapy-induced necrosis is a difference in histopathological interpretation between the centres. This is unlikely to explain such a large difference as the pathologists at both centres adhere to well established international criteria for assessing tumour response.[27] Another possibility is that the surgical intervention took place at different times, i.e., the patients had more neoadjuvant chemotherapy at centre 1 than at centre 2. The range of times to definitive surgery were, however, not significantly different between the two centres. We believe that the difference between the two chemotherapy regimes employed at the two centres offers the most likely explanation for the differences in chemotherapy necrosis.

*Chemotherapy regimes*

The chemotherapy regimes used at the two centres differed significantly in that the regimes used at Centre 1 involved three- or four-drug regimes (involving a combination of doxorubicin, cisplatin, methotrexate and ifosfamide), whilst the majority of patients at Centre 2 usually had a two-drug regime (principally doxorubicin + cisplatin). A total of 73% of patients at Centre 1 had a good (> 90% necrosis) result compared with only 29% at centre 2. This difference is dramatic and would appear to indicate the supremacy of a multidrug regime although previous randomised studies have failed to show this.[1] We cannot exclude, however, the possibility that some other factor such as dose intensity may be responsible for this difference although we feel this is not likely.

It is interesting to note that a good response is as effective in both centres; that is to say, if the tumour is sensitive to the chemotherapeutic agents then, after a period of 300 weeks, appearance of metastatic disease or local recurrence is highly unlikely. In addition, this also shows that the follow-up policy and subsequent imaging techniques used are equally effective in both centres. There is no reason to suggest that centre 1 is more effective at picking up metastatic disease in the pre-treatment setting creating a falsely effective treatment technique when compared to centre 2 as both centres used similar staging techniques with CT chest and bone scans prior to treatment.

A poor response to initial chemotherapy led to a detrimental effect on patient survival at both centres, but the effect was more apparent at Centre 2. The reasons for this were not investigated as part of this study. It is possible that second line chemotherapy may 'rescue' more patients at Centre 1 than at Centre 2.

Our study identifies tumour volume, percentage chemotherapy-induced necrosis and alkaline phosphatase as major patient prognostic indicators for DFS and OS. A combination of raised alkaline phosphatase and poor chemotherapy induced necrosis are the most accurate predictors of poor DFS and OS. The predictive powers of individual prognosticators hold different values in each cohort. Chemotherapy regime used in each centre is a potential major factor in explaining apparent survival differences in patients treated for conventional osteosarcoma. However, this is a retrospective study and a large randomised prospective study could help resolve these issues. In any case, the rarity of these tumours makes continued international co-operation between specialist centres essential if we are to maximise treatment potential.

### Acknowledgements

This study was kindly supported by grants from the following organisations: The Cancer Research Campaign; Friend's Of Rosie Children's Charity; The Royal Orthopaedic Hospital, Birmingham, UK.

### References

1. Souhami RL, Craft AW, Van der Eijken, *et al.* Randomised trial of two regimes of chemotherapy in operable osteosarcoma: a study of the European Osteosarcoma Intergroup. *Lancet* 1997; 350: 911–7.
2. Provisor AJ, Ettinger LJ, Nachman JB, *et al.* Treatment for non-metastatic osteosarcoma of the extremity with preoperative and postoperative chemotherapy: a report from the Children's Cancer Group. *J Clin Oncol* 1997; 15: 6–84.
3. Bacci G, Ferrari S, Bertoni F, *et al.* Long-term outcome for patients with non-metastatic osteosarcoma of the extremity treated at the Istituto Ortopedico Rizzoli according to the Istituto Ortopedico Rizzoli/osteosarcoma-2 protocol: an updated report. *J Clin Oncol* 2000; 18: 4016–27.
4. Winkler K, Beron G, Kotz R, *et al.* Neoadjuvant chemotherapy for osteogenic sarcoma: results of the Cooperative German/Austrian study. *J Clin Oncol* 1984: 2: 617–24.
5. Saeter G, Wiebe T, Wiklund T, *et al.* Chemotherapy in osteosarcoma the Scandinavian Sarcoma Group experience. *Acta Orthop Scand* 1999; 70 (Suppl 285): 74–82.
6. Bacci G, Ferrari S, Mercuri M, *et al.* Neoadjuvant chemotherapy for extremity osteosarcoma: preliminary results of the Rizzoli's 4th study. *Ann Oncol* 1998; 37: 41–8.
7. Whelan JS. Paediatric update: Osteosarcoma. *Eur J Cancer* 1997; 33: 1611–9.
8. Saeter G, Elomaa I, Wahlqvist Y, *et al.* Prognostic factors in bone sarcomas. *Acta Orthop Scand* 1997; 68 (Suppl 273): 156–60.
9. Davis AM, Bell RS, Goodwin PJ. Prognostic factors in osteosarcoma: a critical review. *J Clin Oncol* 1994; 12: 423–31.
10. Glasser DB, Lane JM, Huvos AG, *et al.* Survival, prognosis and therapeutic response in osteogenic sarcoma. *Cancer* 1992; 69: 698–708.
11. Bacci G, Picci P, Ferrari S, *et al.* Prognostic significance of serum alkaline phosphatase measurements in patients with osteosarcoma treated with adjuvant or neoadjuvant chemotherapy. *Cancer* 1993; 71(4): 1224–30.
12. Hudson M, Jaffe MR, Jaffe N, *et al.* Pediatric osteosarcoma: therapeutic strategies, results, and prognostic factors derived from a 10-year experience. *J Clin Oncol* 1990; 8: 1988–97.
13. Bielack SS, Kempf-Bielack B, Delling G, *et al.* Prognostic factors in high-grade osteosarcoma of the extremities or trunk: an anlysis of 1702 patients treated on neoadjuvant cooperative osteosarcoma study group protocols. *J Clin Oncol* 2002; 20: 776–90.
14. Bacci G, Picci P, Ferrar S, *et al.* Primary chemotherapy and delayed surgery for nonmetastatic osteosarcoma of the extremities. Results in 164 patients preoperatively treated with high doses of methotrexate followed by cisplatin and doxorubicin. *Cancer* 1993; 72: 3227–38.
15. Grimer RJ, Taminiau AM, Cannon SR. Surgical outcomes in osteosarcoma. *J Bone Joint Surg* 2002; 84-B: 395–400.
16. Bramwell VHC, Burgers M, Sneath R, *et al.* comparison of two short intensive intensive adjuvant chemotherapy regimens in operable osteosarcoma of limbs in children and young adults: the first study of the European osteosarcoma Intergroup. *J Clin Oncol* 1992; 10: 1579–91.
17. Statview. Berkely, CA. Abacus Concepts, 1996.
18. Bacci G, Ferrari S, Lari S, *et al.* Osteosarcoma of the limb. *J Bone Joint Surg* 2002; 84B: 88–92.
19. Weeden S, Grimer RJ, Cannon SR, Taminiau AM, Uscinska BM. The effect of local recurrence on survival in resected osteosarcoma. *Eur J Cancer* 2001; 37: 39–46.
20. Picci P. Osteosarcoma: relationship of response to preoperative chemotherapy and type of surgery to local recurrence. *J Clin Oncol* 1994; 12: 2699–705.
21. Picci P, Sangiorgi L, Bahamonde L, *et al.* Risk factors for local recurrence after limb-salvage surgery for high grade osteosarcoma of the extremities. *Ann Oncol* 1997; 8: 899–903.
22. Bacci G, Ferrari S, Mercuri M, *et al.* Predictive factors for local recurrence in osteosarcoma: 540 patients with extremity tumours followed for minimum 2.5 years after neoadjuvant chemotherapy. *Acta Orthop Scand* 1998; 69: 230–6.
23. Spanier SS, Shuster JJ, Vander Griend RA. The effect of local extent of the tumour on prognosis in osteosarcoma. *J Bone Joint Surg* 1990; 72A: 643–53.
24. Hauben EI, Weeden S, Pringle J, Van Marck EA, Hogendoorn PC. Does the histological subtype of high grade central osteosarcoma influence the response to treatment with chemotherapy and does it affect overall survival? A study on 570 patients of two consecutive trials of the European Osteosarcoma Intergroup. *Eur J Cancer* 2002; 38: 1218–25.
25. Taylor WF, Ivins JC, Pritchard DJ, *et al.* Trends and variability in survival among patients with osteosarcoma: a 7-year update. *Mayo Clin Proc* 1985; 60: 91–104.
26. Rosen G, Tau L, Sanmaneechai, *et al.* Rationale for multi-drug chemotherapy in the treatment of osteogenic sarcoma. *Cancer* 1975; 35: 936–45.
27. Sulzer-Kuntschik M, Delling G, Beron G, Sigmund R. Morphological grades of regression in osteosarcoma after polychemotherapy – study COSS 80. *J Cancer Res Clin Oncol* 1983;106 (Suppl): 21–4.
